# An unusually high prevalence of allergic rhinitis at high altitudes in 6–7 year old children – An epidemiological study^☆^

**DOI:** 10.1016/j.waojou.2024.100887

**Published:** 2024-05-04

**Authors:** Ying-Qin Gao, Jun Jie Seah, Mei-Lan Wang, Qing-ping Tang, De-Yun Wang, Xian-Yun Bi, Hua-wei Han, Tie-Song Zhang, Jing Ma

**Affiliations:** a Department of Otolaryngology, Head and Neck Surgery, Kunming Children's Hospital, Kunming, China; b Department of Otolaryngology, Yong Loo Lin School of Medicine, National University of Singapore, Singapore, Singapore; c Infectious Diseases Translational Research Programme, Yong Loo Lin School of Medicine, National University of Singapore, Singapore, Singapore

**Keywords:** Altitude, Hypersensitivity, Rhinitis, Child

## Abstract

**Objectives:**

To compare the epidemiology and disease patterns of allergic rhinitis (AR) at 2 different altitudes in children aged 6–7 years, and subsequently to compare with and augment data from international studies.

**Materials and methods:**

This is a multistage, clustered and stratified random sample study. The study area comprises 2 distinct areas within Yunnan Province, China. Low altitude was represented by Xishuangbanna Prefecture (XB), while high altitude was represented by Diqing Prefecture (DiQ). Each study area was subdivided into 3 sub-areas, and children aged 6–7 years were randomly sampled based on proportion-weighted sampling. The area studied includes the well-known area of Shangri-La city. Questionnaires were distributed and jointly completed by study participants and their parents or guardians, under the guidance of professional medical staff.

**Results:**

2796 valid questionnaires out of 2933 distributed were obtained (survey response rate 95.3%). The prevalence of AR is statistically significantly higher at high altitude (DiQ, 36.0%, 95%CI 33.2–38.8) as compared to low altitude (XB, 19.7%, 95%CI 17.8–21.6) (p < 0.001). Both areas studied had a greater prevalence of AR compared to international data. In both XB and DiQ, male gender, history of early antibiotic use, urban place of birth and place of residence, presence of smokers within the same household, family history of allergic diseases (such as atopic dermatitis), as well as higher parental educational level were all associated with a higher prevalence of AR (p < 0.05). In DiQ, the prevalence of AR in Han ethnicity was greater than that of ethnic minorities (p < 0.05). In XB, being a single child was associated with an increased prevalence of AR compared to those who had siblings (p < 0.05).

**Conclusion:**

Our study found that the prevalence of AR is relatively greater at higher altitudes. Genetic and environmental factors both play an important role in the pathogenesis of AR. While altitude may be an important environmental factor, confounding factors may include humidity, temperature and distribution pattern of common aeroallergens.

## Introduction

Allergic rhinitis (AR) is a common disease of childhood which is characterized by sneezing, rhinorrhea, nasal itch, and nasal obstruction. It has a negative impact on quality of life and productivity, thereby representing a sizable healthcare burden on society. The incidence of AR is rising year by- year, with an estimated prevalence of 500 million affected globally.^1^ Research from the International Study of Asthma and Allergies in Childhood (ISAAC) revealed that the prevalence of AR in 6–7 year-old children is 8.5%, which increases at a rate of 0.17% annually.^2^ Research has also shown that AR is the commonest chronic paediatric disease, affecting up to 40% of children.^1^

The pathogenesis of AR is multifactorial, and is commonly thought to comprise both genetic and environmental factors. Common allergens responsible for AR include house dust mites (HDMs) such as *Dermatophagoides pteryonissinus*, *Dermatophagoides farniae*, and *Blomia tropicalis*, dog and cat epithelia, cockroach, and grass pollens, which are known to be influenced by geographic and climate factors.

Throughout the years, while many efforts have been made to investigate the epidemiology of AR, data pertaining to the paediatric population, especially at high altitudes, are considered to be underreported.^3^ The geographical landscape of China is vast and varied, and encompasses areas of varying altitudes, thus presenting a prime opportunity to investigate the heterogenous population within. In order to understand the factors influencing AR in schoolgoing children, we investigated the prevalence of AR and patient characteristics of children aged 6-7 year-old from 2 distinct regions within Yunnan Province with contrasting altitudes. The primary aim of this study was to determine if geographical altitude had an association with the prevalence of AR, in children aged 6–7 years. In addition, we also aim to compare our data with internationally available studies which are part of the International Study of Asthma and Allergies in Children (ISAAC) study, and contribute to the existing knowledge pool on the topic of allergic diseases.

## Materials and methods

### Survey area and population

This is a multistage, clustered and stratified random sample study of children aged 6–7 years of age. The study area comprises 2 distinct geographical locations within Yunnan Province, namely Xishuangbanna (XB) and Diqing (DiQ) Prefectures. XB is located in the south of Yunnan Province, with an average altitude of 800 m above sea level, which includes Jinghong City (JH), Menghai County (MH) and Mengla County (ML). In contrast, DiQ is located in the northern part of Yunnan Province, and boasts a much higher average altitude of 3245 m above sea level, which includes Shangri-La City (SL), Weixi County (WX) and Deqin County (DeQ). These areas of interest encompass in total approximately over 7° of latitude (from 21°8′ N to 29°15′ N) and over 8° of longitude (from 97°31′ E to 106°11′ E) (Supplementary Fig. A). In 2021, XB has a population size of 1,306,000, as compared to DiQ with a population of 389,000.

The study duration took place over the months of April to May of 2021. Study participants were schoolgoing children aged 6–7 years, who have been residents of Yunnan Province for at least 1 year. We chose this age group where children first embark on formal primary education as most allergic diseases would have manifested by this age, as well as to allow for comparison with internationally available data. Rural and urban residents were selected equally in a 1:1 ratio. Subjects were selected based on the stratified random sampling method. Firstly, an initial study on first grade school children aged 6–7 years in XB and DiQ found that the incidence of allergic disease was estimated at 15% and 25% respectively. Based on the sample size formula: *n* $=\frac{z_{\alpha /2}^{2}\left(1-P\right)}{\varepsilon^{2}P}$, in order to achieve a significance level of 0.05 (⍺) and a 0.15p error tolerance, a sample size of at least 968 participants would be required for XB. To account for buffer in case questionnaires were non-eligible, a sample size of 1070 participants (110%) was selected. Using cluster sampling, the design effect is 1.5, and the minimum sample size is 1460 people. For DiQ, in order to achieve the 0.05 significance level (⍺) and 0.25p error tolerance, the minimum sample size required is 513. Similarly to account for buffer, a sample size of 570 (110%) was selected. Using cluster sampling, the design effect is 1.5, and the minimum sample size is 770 people.

After establishing the minimum sample size required for both XB and DiQ, a multistage sampling protocol was adopted, and the 2 regions were divided into 3 areas according to the distance from the administrative center of the prefecture. The areas included the central, north, and south regions. The target minimum number of students to be sampled from each county were based on the proportion of first-year students (aged 6–7 years) in each school. Only schools with 50 or more first-grade students were included. In total, we aimed to sample at least 679, 393 and 388 students from JH, MH and ML respectively, and 345, 330 and 95 students from SL, WX, and DeQ respectively.

The study protocol was approved by the local Medical Ethics Committee. Before the commencement of the study, the research team met with local government and health officials in the 6 sites of both survey areas to discuss the study. Potential subjects who lived in the survey area for at least 1 year were randomly selected. Referral letters were sent to each school and their parents, and telephone or verbal reminders were conducted by the study coordinator or local medical officials. All subjects were accompanied by their parents or guardians and informed consent was obtained.

### Questionnaire implementation

We designed our questionnaire in collaboration with the Allergy Department of a local Medical College Hospital, and the local Ministry of Science and Technology. We also collaborated with the pulmonology and gastroenterology departments from a local Children's Hospital to further tailor the questionnaire to local conditions. A sample of the questionnaire can be found in **Annex A**.

The questionnaire consists of 2 parts. The first part surveys basic demographic data and factors related to allergic diseases. The second part screens for AR, including symptoms related to AR and non-AR, and the investigation of other comorbid diseases such as asthma, allergic conjunctivitis (AC), food protein allergy, and atopic dermatitis (AD). The questionnaire was jointly completed by study participants and their parents or guardians, under the guidance of professional medical staff.

### Diagnostic criteria

#### Allergic rhinitis

We based our diagnosis of AR on criteria set out in the following publications: Allergic Rhinitis and its Impact on Asthma (ARIA) 2008^1^ and Guidelines for the Diagnosis and Treatment of Allergic Rhinitis 2015, Tianjin (Translated).^4^ AR was diagnosed when at least 2 of the following symptoms were present when associated with exposure to aeroallergens: (1) Paroxysmal sneezing, (2) Nasal discharge, (3) Nasal itch, and (4) Nasal congestion, with symptoms lasting for more than 1 hour each day.

#### Allergic conjunctivitis

The diagnosis of AC was made if the respondent had any one of the following eye symptoms when associated with exposure to aeroallergens: itching, congestion, and tearing.^5^

#### Asthma

The diagnosis of asthma was made if the respondent had been previously diagnosed with asthma by a doctor, or had at least one of the following symptoms in the past twelve months: (1) Wheezing, (2) Cough, (3) Dyspnea, (4) Chest tightness, (5) Shortness of breath, and (6) Nocturnal awakening from breathlessness.^6^

### Statistical methods

Epidata was used for data entry and analysis. Data entry was performed by 2 researchers to reduce errors. Data processing and analysis were performed using SPSS version 17.0. The chi-square (*χ*^2^) test was used to compare the prevalence of AR between groups, where *p* values of less than 0.05 were considered statistically significant. Univariate analysis was firstly performed. Variables which demonstrated statistical significance (p < 0.05) were included as variables in the multivariate analysis. In the multivariate analysis, the dependent variable is whether one suffers from allergic rhinitis, with a value of 1 for those with the disease and 0 for those without the disease. Multivariate analysis was performed using binary logistic regression analysis, where *p* values of less than 0.05 were included in this model.

## Results

### Participants and descriptive data

#### Questionnaire response

**Fig. 1:**
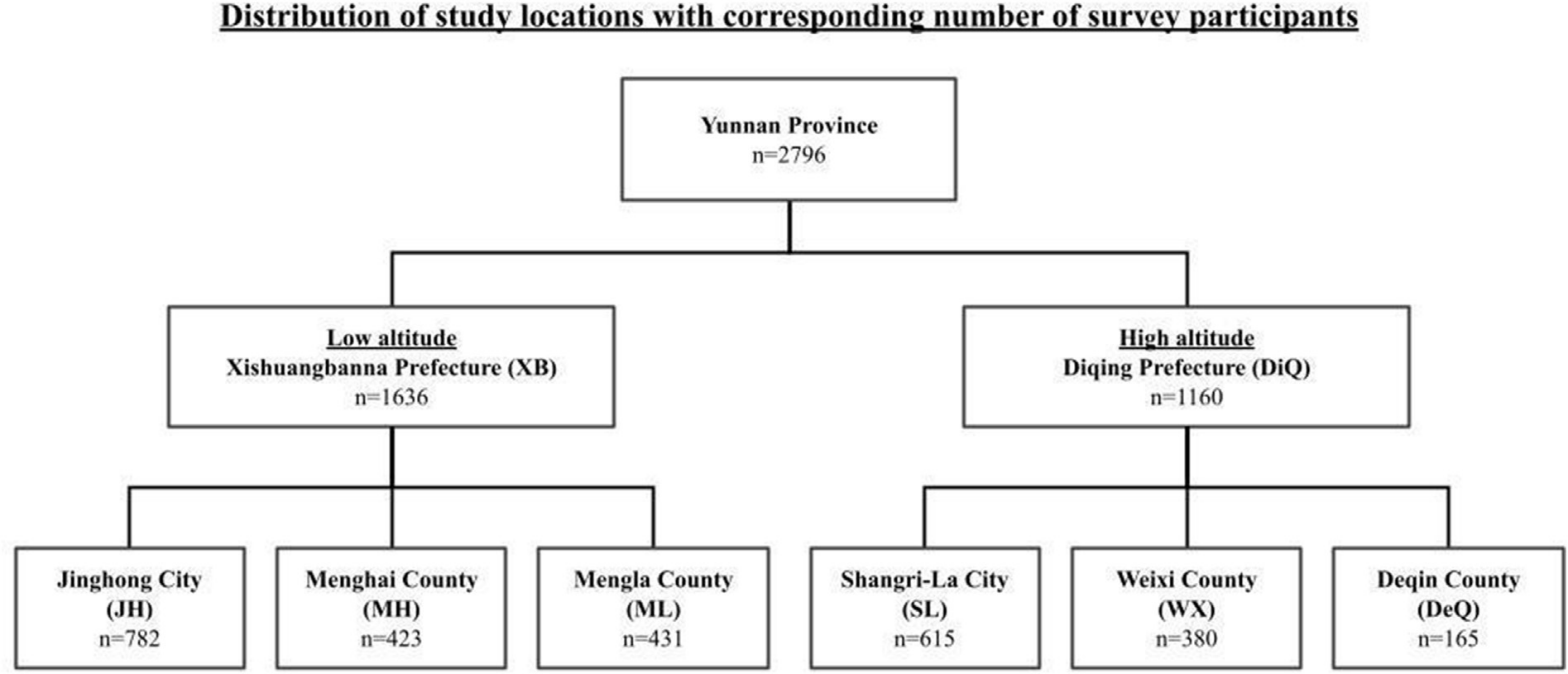
Number of survey participants surveyed by geographical location. Legend: XB: Xishuangbanna Prefecture; DiQ: Diqing Prefecture; JH: Jinghong City; MH: Menghai County; ML: Mengla County; SL: Shangri-La City; WX: Weixi County; DeQ: Deqin County

A total of 2933 questionnaires were distributed and filled in by the participants and their parents or guardians. After excluding invalid questionnaires, 2796 valid questionnaires were obtained (1636 XB, 1160 DiQ), yielding a response rate of 95.3%. The number of people surveyed based on location is shown in Fig. 1. The baseline patient characteristics of our study population are shown in Table 1. The ethnic distribution in XB and DiQ respectively is shown in Supplementary Fig. B.

**Table 1:** Descriptive characteristics of patients sampled from various geographical locations. N: Number of patients.

Variable	Characteristics	XB (Low altitude)	DiQ (High altitude)
		N (%)	N (%)
**Demographic variables**			
Gender	Male	825 (50.4)	586 (50.5)
	Female	811 (49.6)	574 (49.5)
Place of birth	Urban	355 (21.7)	155 (13.4)
	Rural	1281 (78.3)	1005 (86.6)
Place of residence	Urban	370 (22.6)	165 (14.2)
	Rural	1266 (77.4)	995 (85.8)
Family history of smoking	Yes	860 (52.6)	661 (57.0)
	No	776 (47.4)	499 (43.0)
Personal or family history of genetic allergies	Yes	66 (4.0)	27 (2.3)
	No	1570 (96.0)	1133 (97.7)
Early antibiotic use	Yes	246 (15.0)	113 (9.7)
	No	1390 (85.0)	1047 (90.3)
No. of children in family	Single	922 (56.4)	665 (57.3)
	Multiple	714 (43.6)	495 (42.7)
No. living in household	1–3	295 (18.0)	159 (13.7)
	4	558 (34.1)	325 (28.0)
	5	313 (19.1)	226 (19.5)
	6	295 (18.0)	264 (22.8)
	≥7	175 (10.7)	186 (16.0)
Highest parental education	High school and below	1413 (86.4)	938 (80.9)
	College and above	223 (13.6)	222 (19.1)
**Comorbid allergic diseases**			
Allergic conjunctivitis		66 (4.0)	101 (8.7)
Asthma		23 (1.4)	11 (0.9)
Food protein allergy		9 (0.5)	19 (1.6)
Atopic dermatitis		39 (2.3)	41 (3.5)

Legend: XB: Xishuangbanna Prefecture; DiQ: Diqing Prefecture

#### Prevalence of AR

The average self-reported AR prevalence in XB and DiQ collectively was 26.5%. A total of 399 (28.3%) male and 337 (24.3%) female students had AR. Within XB, the prevalence of AR was 323 (19.7%), with 207 (26.5%), 56 (13.2%) and 60 (13.9%) students from JH, MH and ML respectively. Within DiQ the prevalence of AR was 418 (36.0%), with 217 (35.3%), 167 (43.9%), and 34 (20.6%) students from SL, WX, and DeQ respectively. At higher altitudes, DiQ had almost twice the prevalence of AR as compared to the relatively lower altitude area of XB (Supplementary Fig. C).

Perennial AR predominates in both XB and DiQ. Of all the children with AR in XB, 234 (72.45%) had perennial AR and 89 (27.55%) had seasonal AR. Similarly in DiQ, 322 children (79.43%) had perennial AR and 86 children (20.57%) had seasonal AR. This is depicted in Supplementary Fig. D.

#### Ethnicity

A total of 148 children of Han ethnicity had self-reported AR from XB and DiQ collectively. In XB, the prevalence of AR in Han children was 23.2%, as compared to 50.5% in DiQ. The prevalence of AR within minority ethnic groups are as follows: 64 Dai (17.0%), 58 Hani (18.1%), 32 Yi (20.5%), 30 Blang and Lahu (19.6%), 171 Tibetans (33.3%), 106 Lisu (32.2%), 39 Nakhi (37.9%), and 88 (28.4%) were of the remaining 16 ethnic minorities.

#### Comorbid diseases

The prevalence of comorbid allergic diseases, including AC, asthma, food protein allergy, and AD, is depicted in Table 1.

### Statistical analysis

Comparison of the overall prevalence of AR revealed that DiQ had significantly greater prevalence of AR as compared to XB (p < 0.001). Statistical testing is presented in Table 2.

**Table 2:** Statistical analysis comparing prevalence of AR in XB and DiQ Prefectures.

Location	Prevalence of AR (%)	95% Confidence interval (%)	*χ*^2^	*p*
XB	19.7	17.8–21.6	92.482	<0.001
DiQ	36.0	33.2–38.8		

Legend: XB: Xishuangbanna Prefecture; DiQ: Diqing Prefecture

Results of univariate and multivariate analyses are presented in Table 3, and further elaborated in the relevant sections below.

**Table 3:** Results of univariate analysis and multivariate binary logistic regression analysis of various factors in XB and DiQ Prefectures.

Prefecture	Variable	AR prevalence (N [%])	Univariate analysis		Multivariate analysis				
			*χ*^2^	*p*	*b*	SE_b_	Wald *χ*^2^	OR (95%CI)	*p*
**XB (Low altitude)**	**Gender**		1.02	0.313					
	Male	171 (20.7)							
	Female	152 (18.7)							
	**Ethnicity**		5.68	0.339					
	Han	102 (23.2)							
	Dai	64 (17.0)							
	Hani	58 (18.1)							
	Blang/Lahu	30 (19.6)							
	Yi	32 (20.5)							
	Others	37 (19.5)							
	**Location**		42.85	<0.001					
	MH	56 (13.2)			1				
	JH	207 (26.5)			0.84	0.17	24.56	2.31 (1.66,3.21)	**<0.001**
	ML	60 (13.9)			0.06	0.21	0.09	1.06 (0.71,1.59)	0.766
	**Place of birth**		9.93	0.002					
	Urban	91 (25.6)							
	Rural	232 (18.1)							
	**Place of residence**		7.10	0.008					
	Urban	91 (24.6)							
	Rural	232 (18.3)							
	**Family history of smoking**		1.31	0.252					
	No	144 (18.6)							
	Yes	179 (20.8)							
	**Personal or family history of genetic allergies**		39.74	<0.001					
	No	290 (18.5)			1				
	Yes	33 (50.0)			1.20	0.27	19.71	3.33 (1.96,5.66)	**<0.001**
	**Early antibiotic use**		67.93	<0.001					
	No	227 (16.3)			1				
	Yes	96 (39.0)			1.06	0.16	46.26	2.88 (2.13,3.91)	**<0.001**
	**Highest parental education**		9.44	0.002					
	High school and below	262 (18.5)							
	College and above	61 (27.4)							
**DiQ (High altitude)**	**Gender**		5.64	0.018					
	Male	228 (38.9)			1				
	Female	185 (32.2)			0.30	0.13	5.40	0.74 (0.57,0.95)	0.020
	**Ethnicity**		14.67	0.005					
	Han	46 (50.5)			1				
	Naxi	39 (37.9)			0.56	0.31	3.39	0.57 (0.31,1.04)	0.066
	Tibetian	171 (33.1)			0.59	0.24	5.92	0.55 (0.34,0.89)	0.015
	Lisu	106 (32.2)			0.90	0.27	11.16	0.41 (0.24,0.69)	0.001
	Others	51 (42.5)			0.33	0.29	1.24	0.72 (0.41,1.28)	0.266
	**Location**		34.96	<0.001					
	WX	167 (43.9)			1				
	SL	217 (35.3)			0.80	0.16	24.28	0.45 (0.33,0.62)	**<0.001**
	DeQ	29 (17.6)			1.21	0.24	25.64	0.30 (0.19,0.48)	**<0.001**
	**Place of birth**		12.75	<0.001					
	Urban	75 (48.4)			1				
	Rural	338 (33.6)			0.68	0.20	12.28	0.50 (0.34,0.74)	**<0.001**
	**Place of residence**		10.27	0.001					
	Urban	77 (46.7)							
	Rural	336 (33.8)							
	**Family history of smoking**		7.20	0.007					
	No	156 (31.3)			1				
	Yes	257 (38.9)			0.30	0.13	5.15	1.35 (1.04,1.75)	0.023
	**Personal or family history of genetic allergies**		17.84	<0.001					
	No	393 (34.7)			1				
	Yes	20 (74.1)			1.59	0.46	11.81	4.90 (1.98,12.15)	0.001
	**Early antibiotic use**		18.45	<0.001					
	No	352 (33.6)			1				
	Yes	61 (54.0)			0.67	0.21	9.94	1.95 (1.29,2.96)	0.002
	**Highest parental education**		13.95	<0.001					
	High school and below	310 (33.0)							
	College and above	103 (46.4)							

**Legend**: N: Number of children; *χ*^2^: Univariate chi-square test value; *p*: *p*-value of the corresponding chi-square statistic; *b*: Unstandardised regression coefficient; SE: Standard error; Wald *χ*^2^: Multivariate chi-square value; OR: Odds ratio, 95% CI: 95% Confidence interval

#### Univariate analysis of relevant influencing factors

In both XB and DiQ, male gender, history of early antibiotic use, urban place of birth and place of residence, presence of smokers within the same household, family history of genetic allergic diseases (such as AD), as well as higher parental educational level (college and above) were all associated with a higher prevalence of AR. In DiQ, the prevalence of AR in Han ethnicity was greater than that of ethnic minorities. In XB, being a single child was associated with an increased prevalence of AR compared to those who had siblings. The above correlations all individually reached statistical significance (*p* < 0.05).

It was also found that while Yunnan Province is dominated by ethnic minorities, the prevalence of AR in the Han population is much higher than that of other ethnic minorities (*p* < 0.05). The prevalence of AR in the Han population living in XB is 23.2%, compared to that of ethnic minorities at 18.5%, whilst in DiQ, the prevalence of AR in the Han population was 50.5%, compared to 36.3% for ethnic minorities.

#### Multivariate analysis

When variables with *p* < 0.05 based on the univariate analysis were incorporated into a binary logistic regression model, it revealed that children who had a family history of allergy had approximately 5 times the odds of developing AR compared to children who did not have a family history of allergy (OR 4.904, 95%CI 1.980–12.148). It was also found that early antibiotic use was associated with approximately twice the odds of developing AR compared to no early antibiotic use (OR 1.953, 95%CI 1.288–2.960) (Table 3).

## Discussion

### Prevalence of AR

Our study found that the prevalence of AR was statistically significantly greater at high altitude (DiQ) as compared to low altitude (XB). When comparing with existing data, our study also found that the self-reported prevalence of AR in both XB and DiQ (19.7% and 36.0% respectively) were significantly higher than the international prevalence of AR, which is estimated to be about 8.5–14.6%,^7^ and also significantly higher than the average prevalence of eight cities in China, estimated to be about 9.8%.^8^ This holds true even when comparing our data to research on children of similar age group, where an ISAAC phase III study found that the international prevalence of rhinoconjunctivitis symptoms in children aged six to seven was 8.5%.^9^ While our study compared geographical areas of “high” and “low” altitude, even the low altitude area of XB was elevated approximately 800 m above sea level on average, which could still be relatively higher in altitude compared to other international areas.

Our study found that the prevalence of AR in DiQ (high altitude) was almost twice that of XB (low altitude). Contrastingly, previous studies showed that high altitude had a beneficial effect on allergic rhinoconjunctivitis^10^ and other atopic diseases, such as asthma.^11^^,^^12^ Possible reasons that have been put forward to account for these findings could include reduced HDM and pollen concentrations, reduced air pollution, as well as reduced humidity at high altitudes.^13^ HDMs and other aeroallergens such as mould are known to thrive in moist humid environments.^14^, ^15^, ^16^ In our study area however, the average annual humidity level is 73.34% and 79.19% at XB and DiQ, respectively, which is considered humid. Chen et al investigated the distribution of *Dermatophagoides* mites in various areas in China, and found that increased humidity positively correlated with *Dermatophagoides* sensitization in AR patients.^17^ Humidity and climate may hence be a potential confounder when analysing the association between altitude level and the prevalence of AR.

From a pathomechanistic perspective, one study hypothesized that hypoxia experienced at high altitudes reduces the levels of T-lymphocytes and consequently local airway inflammation.^18^ Another study suggested that atrial natriuretic peptide (ANP) may play a role in the regulation of bronchial tone, and increased ANP production at high altitudes contributed to decreased bronchial responsiveness in their subjects.^19^ However, both these studies were performed in patients with asthma. While AR may be related to asthma via its links to atopy and type 2 inflammation, the underlying disease mechanisms are unlikely identical and would benefit from further studies before extrapolations are made.

On the other hand, several studies in the literature seem to corroborate with our data. Charpin et al compared schoolchildren living at high altitudes in the Alps against schoolchildren living at sea level, and found that the prevalence of hay fever with a positive skin prick test (SPT) to grass pollens was statistically significantly higher in the high altitude group.^20^ There have also been studies that demonstrated the lack of benefit of high altitude on allergic respiratory disease. HDM allergen concentrations did not significantly change with increasing altitude ranging from 400 m to 2600 m above sea level,^21^ and demonstrated high allergen levels even in areas as high as 2500 m above sea level.^22^ Based on the general clinical records of SPT results from both XB and DiQ (unpublished data), it was found that sensitization to *Dermatophagoides pteronyssinus* and *Dermatophagoides farinae* is 54.81% and 52.64% in XB, and 26.31% and 28.68% in DiQ, respectively. Sensitization to other pollen allergens, such as from Rapeseed (*Brassica* spp.), Sagebrush (*Artemisia sieversiana*), Sunflower, *Populus* tree, Chinese Parasol tree (*Firmiana simplex*), Chinese ash tree (*Fraxinus chinensis*), Chinese juniper (*Juniperus chinensis*) are found in both areas ranging from approximately 10-30%.

As can be seen, the existing literature pertaining to the effect of high altitude on the prevalence of AR and atopic diseases in general is divided, suggesting that the pathogenesis of AR is complex and multifactorial in nature. Further, it may become more apparent that AR is not only affected by aeroallergen exposure, but could also possibly be influenced by locoregional geographical factors such as local altitude, climate, vegetation, ethnicity and other factors. The heterogeneity in climate and environmental conditions therefore poses a barrier towards the generalisability of study findings on this topic.

### Perennial AR vs seasonal AR

While perennial AR predominates in both XB and DiQ, DiQ had a larger proportion of perennial AR as compared to XB. This could be attributed to the specific allergens present that cause disease. Various studies showed higher concentrations of perennial allergens at higher altitude, such as that of HDM^21^^,^^22^ and airborne fungal allergens.^23^ Seasonal AR is conventionally associated with allergens such as certain pollens, which are affected by variations in the climate and wind pollination seasons. On the other hand, perennial AR tends to be caused by allergens that are less affected by climate – namely HDM, pet dander and certain moulds.^24^ While not investigated within the scope of this study, the authors propose that the marginally greater proportion of perennial AR in DiQ could be attributed to a greater proportion of perennial allergens, such as HDM. Further studies would however be required to delineate the allergen distributions and patterns at various altitudes.

### Ethnicity

Interestingly, children of Han ethnicity had higher proportion of AR as compared to minority ethnic groups, regardless of high or low altitude. The predominance of AR in Han ethnicity as compared to ethnic minorities is in keeping with a recent meta-analysis comprising data from 2001 to 2021.^25^ A few genetic studies have been done that can support this finding. Mou et al found that polymorphisms in the TIM-1 gene is associated with AR susceptibility in a Han Chinese population.^26^ Another study by Hu et al found IL-23 R polymorphisms in a Chinese Han population that are associated with susceptibility to AR.^27^ Deng et al found that polymorphisms in surfactant protein A was associated with AR in a Han population.^28^ Yet, while this held true regardless of high or low altitude, the difference in proportion between Han and ethnic minorities with AR is greater in DiQ (50.5% Han, 36.3% minority), as opposed to XB (23.2% Han, 18.5% minority). This further supports the theory that AR develops via a complex interaction between genetic and environmental factors, of which altitude and climate could be plausible factors.

### Comorbid diseases

AR has long been associated with atopy and other allergic diseases. The basis for atopy may stem from an exaggerated Th2 response involving cytokines such as IL-4, IL-5, and IL-13, as well as eosinophilia. Our study found that the proportion of children with comorbid allergic diseases was uniformly greater in DiQ, with the exception of asthma, which had a greater proportion in XB.

One plausible reason could be attributed to allergic diseases being triggered by similar antigens. This could account for why the proportion of comorbid AC in DiQ is more than twice that of XB, since similar allergens tend to trigger both AR and AC, to the extent that they are sometimes referred to as a common entity – allergic rhinoconjunctivitis. Common allergens include HDM, cockroaches and pollen.^29^ With a greater prevalence of AR at higher altitude there is also a greater prevalence of comorbid AC, as compared to low altitude.

Food protein allergy and AD may not share the same extent of overlap as AC. Food protein allergy tends to be triggered by raw fruits and vegetables, or cow's milk, depending on whether the allergy is IgE or non-IgE mediated.^30^ AD is commonly triggered by food allergens (eg, peanut, eggs), infections or even emotional stress.^31^ This may explain why the discrepancy in proportion of children with food protein allergy and AD is smaller.

Yet, other studies have shown that dysbiosis of the gut flora may underscore the mechanism by which atopy develops. Under the “hygiene hypothesis” originally described by Strachan,^32^ children who are exposed to a broad range of environmental substances are purported to develop a more robust and diverse microbiota, with consequently reduced susceptibility to atopic diseases.^33^ Indeed, gut dysbiosis has been shown to be related to the development of atopic diseases such as asthma and AR.^34^ The authors propose that while the initiating trigger allergen may differ, there may be a common pathway that eventually leads to an exaggerated Th2 inflammation, manifesting phenotypically as atopy.

### Multivariate analysis

Our multivariate analysis found that children with a family history of allergy was statistically significantly associated with a higher odds of developing AR. This supports the hypothesis that the pathogenesis of AR has a genetic or inheritable component, similarly seen in many other atopic diseases such as asthma.^35^ While studies attempt to elucidate a link between environmental factors such as altitude on the pathogenesis of AR, genetic and heritable factors still play an integral role.

It is interesting to note that early antibiotic use has approximately twice the odds of developing AR. Our findings echo that of a Japanese study which found that antibiotic use within the first 2 years of life was a risk factor for current asthma, AD, and AR in 5 year-old children.^36^ Another study by Alm et al. showed that antibiotic use in the first week of life increased the risk of AR at school age.^37^ Referencing the “hygiene hypothesis” once again, early use of antibiotics may disrupt the diversity and makeup of the infant gut flora, which has been shown to be related to an increased risk of developing allergic diseases.^38^ This sheds further light on the underlying disease processes of AR.

## Limitations

While best effort has been made to ensure the robustness of our study, it is prone to several limitations. Firstly, our study takes on a cross-sectional design, which can only offer information over a snapshot of time. Dynamic changes such as trends and changes in disease patterns cannot be assessed, which may leave out valuable insights. Nonetheless, the information gathered can contribute to the existing knowledge pool of what is currently understood about AR, and act as a stepping stone for further studies.

Secondly, the diagnosis of AR in our study is made clinically based on a questionnaire. Facilities for objective testing such as SPT was not feasible due to accessibility issues as well as COVID-19 restrictions. However, the questionnaire was validated by reputable local healthcare authorities, and we collaborated with local healthcare professionals to ensure that it was tailored to our study population. In addition, objective allergy testing is not compulsory in the presence of typical signs and symptoms, especially in the primary care or rural setting where facilities are limited. Empirical trial of treatment is usually sufficient to address bothersome symptoms.

Thirdly, the usage of a questionnaire leaves us vulnerable to recall bias, which is part and parcel of a survey method. To mitigate this issue, the questionnaire was jointly completed by both the child and parent or guardian, where the benefit of corroboration would ideally reduce the extent of recall bias.

On this topic, future studies can consider a longitudinal study design with incorporation of objective testing such as SPT or *in vitro* allergy testing. With this information, the diagnosis of AR is made clearer and patients can be characterized based on the specific allergen they are sensitized to. Other future studies could also consider characterizing the profile of allergens at various altitudes, which could provide correlation to the patterns of AR seen at various geographical locations.

## Conclusion

In conclusion, we found that the self-reported prevalence of AR in children aged six to seven years old at different altitudes in Yunnan Province was high and varied. The prevalence of AR at high altitudes was almost twice as compared to low altitudes. The prevalence of AR in Han children was higher than that of other ethnic minorities. Future research studies on the topic of AR can consider more in-depth analyses and encompass larger geographical regions to overcome the shortfalls of this study, with the eventual aim of establishing a standardized diagnostic evaluation and treatment for AR in local schoolgoing children.

## Abbreviations

AC, Allergic conjunctivitis; AD, Atopic dermatitis; ANP, Atrial natriuretic peptide; AR, Allergic rhinitis; ARIA, Allergic Rhinitis and its Impact on Asthma; CI, Confidence interval; DeQ, Deqin County; DiQ, Diqing Prefecture; Ig, Immunoglobulin; ISAAC, International Study of Asthma and Allergies in Childhood; JH, Jinghong City; MH, Menghai County; ML, Mengla County; OR, Odds ratio; SL, Shangri-La City; WX, Weixi County; XB, Xishuangbanna Prefecture.

## This project received the following funding

This research project is supported by (1) the Basic Research and Key Research and Development Plan of Yunnan Province, China (202103AF140008) and (2) Joint Special Fund Project of Yunnan Provincial Science and Technology Department-Kunming Medical University, China (202001AY070001-272).

## Data availability statement

The data that support the findings of this study are available from the corresponding authors, T.S.Z. and J.M. upon reasonable request.

## Author contributions

All authors conceptualized the study; Y.Q.G. and J.J.S. prepared the original draft and are designated co-first authors; M.L.W., Q.P.T., D.Y.W., X.Y.B. and H.W.H. collected and analyzed the data, reviewed and edited the final manuscript; T.S.Z. and J.M. supervised the study. All authors have read and agreed to the published version of the manuscript.

## Ethics approval

The study protocol was approved by the Medical Ethics Committee of the Children's Hospital of Yunnan Province and Kunming [No. 20190822004].

## Authors’ consent for publication

All authors consent to the publication of this manuscript.

## Declaration of competing interest

All authors have no conflict of interest to declare.

## Figures and Tables

**Fig.**
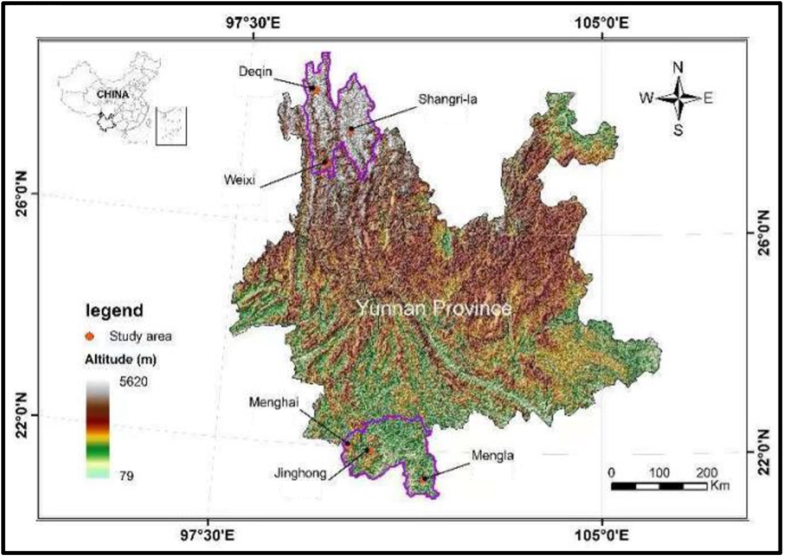
AGeographical map of Yunnan Province, with study areas labelled

**Fig.**
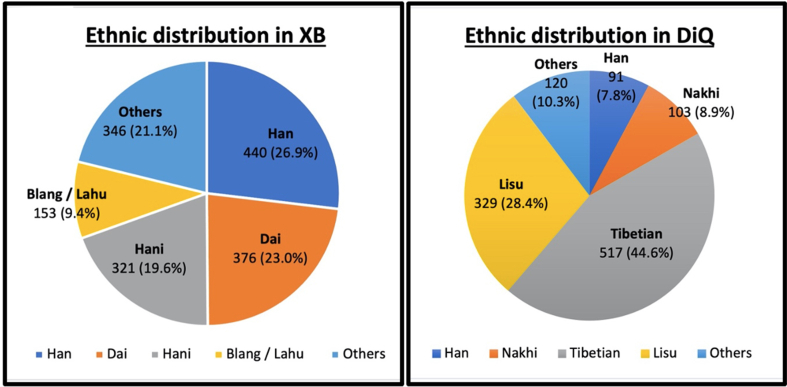
BDistribution of various ethnic groups in XB and DiQ respectively. Legend: XB: Xishuangbanna Prefecture; DiQ: Diqing Prefecture

**Fig.**
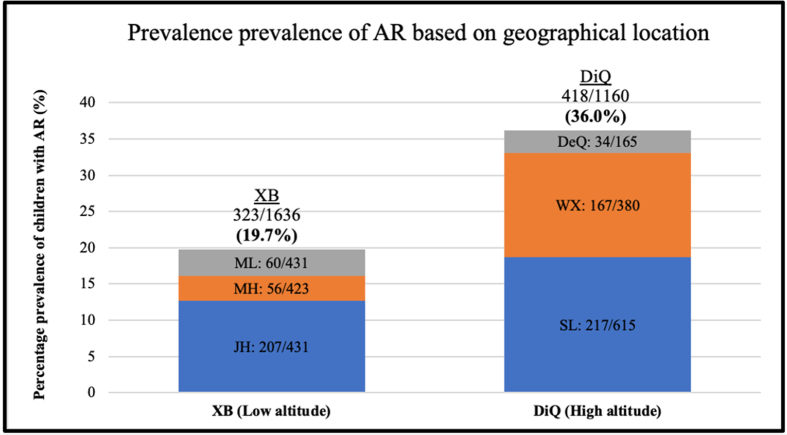
CPercentage prevalence of AR based on geographical location. Legend: XB: Xishuangbanna Prefecture; DiQ: Diqing Prefecture; JH: Jinghong City; MH: Menghai County; ML: Mengla County; SL: Shangri-La City; WX: Weixi County; DeQ: Deqin County

**Fig.**
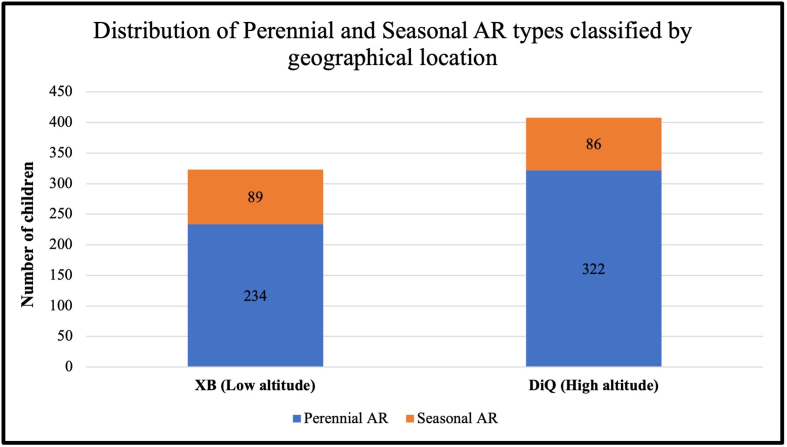
DDistribution of perennial and seasonal AR types classified by geographical location. Legend: XB: Xishuangbanna Prefecture; DiQ: Diqing Prefecture
