# Supplementary material for: An unusually high prevalence of allergic rhinitis at high altitudes in 6–7 year old children – An epidemiological study
Source: World Allergy Organ J. 2024 May 4;17(5):100887. doi: 10.1016/j.waojou.2024.100887 (PMC11089395; doi:10.1016/j.waojou.2024.100887)
Supplement: Multimedia component 1 [file mmc1.docx]

**Annex A: Sample survey form distributed to study participants.**

| **Questionnaire on major allergic diseases of children in Yunnan** | | |
| --- | --- | --- |
| Registration number:  Full name:  Date of birth:  Date of survey response:  Place of residence: | | Ethnicity:  Parity:  Method of contact:  No. living in same household:  Monthly household income: |
| **SECTION 1: DEMOGRAPHIC INFORMATION** | | |
| 1.1 | Gender | Male  Female |
| 1.2 | Place of birth | Urban  Rural |
| 1.3 | Place of residence | Urban  Rural |
| 1.4 | Relationship of caregiver to participant | Parent  Others (please state): _______ |
| 1.5 | Highest parental education level | High school and below  College and above |
| 1.6 | Personal or parental history of genetic allergic diseases | Yes  No |
| 1.7 | Smoking history | Maternal smoking during pregnancy  Number of smokers in household: ___  None of the above |
| 1.8 | Early antibiotic use | Yes  No |
| **SECTION 2: SCREENING FOR ALLERGIC RHINITIS** | | |
| 2.1 | In the past one year, did your child, excluding instances when your child had caught a cold, exhibit the following symptoms: | |
| a)  b)  c)  d)  e) | Runny nose  Paroxysmal or violent sneezing  Blocked nose  Itchy nose  Eye redness, Eye itch, Tearing | Yes / No  Yes / No  Yes / No  Yes / No  Yes / No |
| 2.2 | In the past one year, did your child have recurrent symptoms (more than three times, persisting for more than 2 weeks) of: nasal itch, sneezing, runny nose? | Yes  No |
| 2.3 | If you answered “Yes” to any of the questions 2.1 (a – e) or 2.2, in which months do your child’s symptoms usually appear? | Unrelated to season  Related to season; Please state month: _____ |
| 2.4 | How have the abovementioned nasal symptoms (under Q2.1) affected your child’s daily life? | Visual Analogue Scale (VAS)  0: Not at all  10: Extremely affected  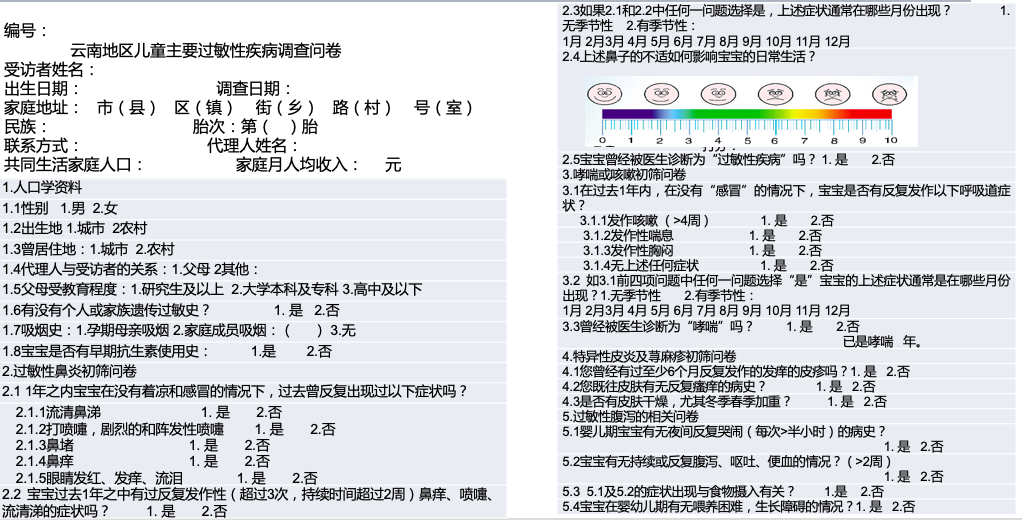 |
| 2.5 | Has your child ever been diagnosed by a doctor with allergic disease? | Yes  No |
| **SECTION 3: SCREENING FOR ASTHMA** | | |
| 3.1 | In the past one year, when your child was not experiencing a cold, did your child have any of the following recurrent respiratory symptoms: | |
| a)  b)  c)  d) | Paroxysmal Cough for more than 4 weeks  Panting  Chest tightness  Not stated above | Yes / No  Yes / No  Yes / No  Yes / No |
| 3.2 | If you answered “Yes” to any of the questions 3.1 (a – e), in which months do your child’s symptoms usually appear? | Unrelated to season  Related to season; Please state month: _____ |
| 3.3 | Has your child ever been diagnosed by a doctor with asthma? | Yes; If yes, state duration since diagnosis: ____  No |
| **SECTION 4: SCREENING FOR ATOPIC DERMATITIS OR URTICARIA** | | |
| 4.1 | Has your child experienced recurrent skin rash for at least 6 months? | Yes  No |
| 4.2 | Has your child experienced recurrent skin itch in the past? | Yes  No |
| 4.3 | Has your child experienced dry skin, especially if exacerbated during winter and spring seasons? | Yes  No |
| **SECTION 5: SCREENING FOR ALLERGIC DIARRHEA** | | |
| 5.1 | During infancy, did your child have a history of recurrent nocturnal crying lasting for at least 30 minutes each episode? | Yes  No |
| 5.2 | Did your child have a history of persistent or recurrent diarrhea, vomiting or blood in stools, lasting for at least 2 weeks? | Yes  No |
| 5.3 | If you answered “Yes” to questions 5.1 and 5.2, were your child’s symptoms related to food intake? | Yes  No  Not applicable |
| 5.4 | During infancy, did your child have a history of feeding difficulty or growth restriction? | Yes  No |
